# DBS: a fast and informative segmentation algorithm for DNA copy number analysis

**DOI:** 10.1186/s12859-018-2565-8

**Published:** 2019-01-03

**Authors:** Jun Ruan, Zhen Liu, Ming Sun, Yue Wang, Junqiu Yue, Guoqiang Yu

**Affiliations:** 1 School of Information Engineering, Wuhan University of Technology, Wuhan, Hubei 430070 China; 2 Department of Electrical and Computer Engineering, Virginia Polytechnic Institute and State University, Arlington, VA 22203 USA; 3 Department of Pathology, Hubei Cancer Hospital, Wuhan, Hubei 430079 China

## Abstract

**Background:**

Genome-wide DNA copy number changes are the hallmark events in the initiation and progression of cancers. Quantitative analysis of somatic copy number alterations (CNAs) has broad applications in cancer research. With the increasing capacity of high-throughput sequencing technologies, fast and efficient segmentation algorithms are required when characterizing high density CNAs data.

**Results:**

A fast and informative segmentation algorithm, DBS (Deviation Binary Segmentation), is developed and discussed. The DBS method is based on the least absolute error principles and is inspired by the segmentation method rooted in the circular binary segmentation procedure. DBS uses point-by-point model calculation to ensure the accuracy of segmentation and combines a binary search algorithm with heuristics derived from the Central Limit Theorem. The DBS algorithm is very efficient requiring a computational complexity of O(n*log n), and is faster than its predecessors. Moreover, DBS measures the change-point amplitude of mean values of two adjacent segments at a breakpoint, where the significant degree of change-point amplitude is determined by the weighted average deviation at breakpoints. Accordingly, using the constructed binary tree of significant degree, DBS informs whether the results of segmentation are over- or under-segmented.

**Conclusion:**

DBS is implemented in a platform-independent and open-source Java application (ToolSeg), including a graphical user interface and simulation data generation, as well as various segmentation methods in the native Java language.

## Background

Changes in the number of copies of somatic genomic DNA are a hallmark in cancer and are of fundamental importance in disease initiation and progression. Quantitative analysis of somatic copy number alterations (CNAs) has broad applications in cancer research [1]. CNAs are associated with genomic instability which causes copy number gains or losses of genomic segments. As a result of such genomic events, gains and losses are contiguous segments in the genome [2]. Genome-wide scans of CNAs may be obtained with high-throughput technologies, such as SNP arrays and high-throughput sequencing (HTS). After proper normalization and transformation of the raw sample data obtained from such technologies, the next step is usually to perform segmentation to identify the regions where CNA occurs. This step is critical, because the signal at each genomic position measured is noisy and the segmentation can dramatically increase the accuracy of CNA detection.

Quite a few segmentation algorithms have been designed. Olshen et al. [3, 4] developed Circular Binary Segmentation (CBS), which relies on the intuition that a segmentation can be recovered by recursively cutting the signal into two or more pieces using a permutation reference distribution. Fridlyand et al. [5] proposed an unsupervised segmentation method based on Hidden Markov Models (HMM), assuming that copy numbers in a contiguous segment have a Gaussian distribution. Segmentation is viewed as a state transition and maximizes the probability of an observation sequence (copy number sequence). Several dedicated HMMs have been proposed [6–8]. Zaid Harchaoui et al. [9, 10] proposed casting the multiple change-point estimation as a variable selection problem. A least-square criterion with a Lasso penalty yields a primary efficient estimation of change-point locations. Tibshirani et al. [11] proposed a method based on a fused Lasso penalty that relies on the L1-norm penalty for successive differences. Nilsen [12] proposed a highly efficient algorithm, Piecewise Constant Fitting (PCF), that is based on dynamic programming and statistically robust penalized least squares principles. By minimizing a penalized least squares criterion, the breakpoints were estimated. Rigaill [13, 14] proposed dynamic programming to retrieve the change-points to minimize the quadratic loss. Yu et al. [15, 16] proposed a segmentation method using the Central Limit Theorem (CLT), which is similar to the idea used in the circular binary segmentation procedure.

Many existing methods show promising performance when the length of an observation sequence is small or moderate to be split. However, as experienced in our own studies, these methods are computationally intensive and segmentation becomes a bottle neck in the pipeline of copy number analysis. With the increasing capacity for raw sample data production provided by high-throughput technologies, a faster algorithm to perform segmentation to identify regions of constant copy numbers is always desirable. In this paper, a novel and computationally highly efficient algorithm is developed and tested.

There are three innovations in the proposed Deviation Binary Segmentation (DBS) algorithm. First, least absolute error (LAE) principle is exploited to achieve high processing efficiency and speed, and a novel integral array-based algorithm is proposed to further increase computational efficiency. Second, a heuristics strategy derived from the CLT helps gaining additional speed optimization. Third, DBS measures the change-point amplitude of mean values of two adjacent segments at a breakpoint. And using the constructed binary tree of significant degree, DBS informs whether the results of segmentation are over- or under-segmented. A central theme of the present work is to build algorithm for solving segmentation problems under a statistically and computationally unified framework. The DBS algorithm is implemented in an open-source Java package named ToolSeg. It provides integrated simulation data generation and various segmentation methods: PCF, CBS (2004), and segmentation method in Bayesian Analysis of Copy Number Mixture (BACOM). It can be used for comparison between methods as well as meeting the needs of the actual segmentation.

## Implementation

### Systems overview

The ToolSeg tool provides functionality for many tasks typically encountered in copy number analysis: data pre-processing, segmentation methods of various algorithms and visualization tools. The main workflow includes: 1) reading and filtering of raw sample data; 2) segmentation of allele-specific SNP array data; and 3) visualization of results. The input includes copy number measurements from single or paired SNP-array or HTS experiments. Allele observations normally need to detect and appropriately modify or filter extreme observations (outliers) prior to segmentation. Here, the median filtering algorithm [17] is used in the ToolSeg toolbox to manipulate the original input measurements. The method of DBS is based on the Central Limit Theorem in probability theory for finding breakpoints and observation segments with a well-defined expected mean and variance. In DBS, the segmentation curves are recursively generated by the recursive splits using the preceding breakpoints. A set of graphical tools is also available in the toolbox to visualize the raw data and segmentation results and to compare six different segmentation algorithms in a statistically rigorous way.

### Input data and preprocessing

ToolSeg requires the raw signals from high-throughput samples to be organized as a one-dimensional vector and stored as a .txt file. Detailed descriptions of the software are included in the Supplementary Material.

Before we performed copy number change detection and segmentation using copy number data, a challenging factor in copy number analysis was the frequent occurrence of outliers – single probe values that differ markedly from their neighbors. Generally, such extreme observations can be due to the presence of very short segments of DNA with deviant copy numbers, technical aberrations, or a combination. Such extreme observations have potentially harmful effect when the focus is on detection of broader aberrations [17, 18]. In ToolSeg, the classical limit filter, Winsorization, is performed to reduce such noise, which is a typical preprocessing step to eliminate extreme values in the statistical data to reduce the effect of possible spurious outliers.

Here, we calculated the arithmetic mean as the expected value $\widehat{\mu}$ and the estimated standard deviation $\widehat{\sigma}$ based on all observations on the whole genome. For original observations, the corresponding Winsorized observations are defined as ${x}_i^{\prime }=f\left({x}_i\right)$, where

1 $$ f(x)=\left\{\begin{array}{c}\widehat{\mu}-\tau \widehat{\sigma},x<\widehat{\mu}-\tau \widehat{\sigma}\\ {}\widehat{\mu}+\tau \widehat{\sigma},x>\widehat{\mu}+\tau \widehat{\sigma}\\ {}\ x,\mathrm{otherwise}\end{array}\right. $$

and *τ* ∈ [1.5, 3], (default 2.5 in ToolSeg). Often, such simple and fast Winsorization is sufficient, as discussed in [12].

### Binary segmentation

Now, we discuss the basic problem of obtaining individual segmentation for one chromosome arm in one sample. The aim of copy number change detection and segmentation is to divide a chromosome into a few continuous segments, within each of which the copy numbers are considered constant.

Let *x*_*i*_, *i* = 1,2, …, *n*, denote the obtained measurement of the copy numbers at each of the *i* loci on a chromosome. The observation *x*_*i*_ can be thought of as a sum of two contributions:

$$ {x}_i={y}_i+{\varepsilon}_i $$

where *y*_*i*_ is an unknown actual “true” copy number at the *i*’th locus and *ε*_*i*_ represents measurement noise, which follows an independent and identically distributed (i.i.d.) with mean of zero. A breakpoint is said to occur between probe *i* and *i* + 1 if *y*_*i*_ ≠ *y*_*i* + 1_, *i* ∈ (1, *n*). The sequence *y*_0_, …, *y*_*K*_ thus implies a segmentation with a breakpoint set {*b*_1_, …, *b*_*K*_}, where *b*_1_ is the first breakpoint, the probes of the first sub-segment are before *b*_1_, the second sub-segment is between *b*_1_ and the second breakpoint *b*_2_, and so on. Thus, we formulated the copy number change detection as the problem of detecting the breakpoint in copy number data.

Consider first the simplest problem of obtaining only one segment. There is no copy number change on a chromosome in the sample. Given the copy number signals of length *n* on the chromosome, *x*_1_, …, *x*_*n*_, and let *x*_*i*_ be an observation produced by independent and identically distributed (i.i.d.) random variable drawn from distribution of expected values given by $\widehat{\mu}$ and finite variances given by ${\widehat{\sigma}}^2$.

The following defines the statistic ${\widehat{Z}}_{ij}$,

2 $$ {\widehat{Z}}_{i,j}=\frac{\sum_{k=i}^{j-1}\left({x}_k-\widehat{\mu}\right)}{\widehat{\sigma}\sqrt{\ j-i}},1<i<j<n+1, $$

where $\widehat{\mu}=\frac{1}{j-i}{\sum}_{k=i}^{j-1}{x}_k$ is the arithmetic mean between point *i* and point *j* (does not include *j*), and $\widehat{\sigma}$ is the estimated standard deviation of *x*_*i*_, $\widehat{\sigma}=\sqrt{\frac{1}{j-i-1}{\sum}_{k=i}^{j-1}{\left({x}_k-\widehat{\mu}\right)}^2}$, which will be discussed later. Furthermore, we define the test statistic

3 $$ \widehat{Z}=\underset{1<i<j<\mathrm{n}+1,j-i>{n}_0\ }{\max}\left|{\widehat{Z}}_{i,j}\right| $$

where *n*_0_ is a pre-determined parameter of the minimum length of CNA.

According to the central limit theorem (CLT), as the sample size (the length of an observation sequence, *j* − *i*) increases to a sufficiently large number, the arithmetic mean of independent random variables will be approximately normally distributed with mean μ and variance *σ*^2^/(*j* − *i*), regardless of the underlying distribution. Therefore, under the null hypothesis of no copy number change, the test statistic ${\widehat{Z}}_{ij}$ asymptotically follows a standard normal distribution, *N* (0, 1). Copy number change segments measured by high-throughput sequencing data usually span over hundreds, even tens of thousands, of probes. Therefore, the normality of ${\widehat{Z}}_{ij}$ is approximated with high accuracy.

Here, let *θ* be a predefined significance level,

4 $$ \wp \left({\widehat{Z}}_{ij}\right)=1-\sqrt{\frac{2}{\pi }}{\int}_{-\infty}^{{\widehat{Z}}_{ij}}{e}^{-\frac{x^2}{2}} dx>\theta $$

We iterate over the whole segment to calculate the *P*-value of $\widehat{Z}$ using the cumulative distribution function of *N* (0, 1). If the P-value is greater than θ, then we will consider that there is no copy number change in the segment. In other words, $\widehat{Z}$ is not far from the center of the shape of the standard normal distribution.

Furthermore, we also introduce an empirical correction to θ which is divided by *L*_*i*, *j*_ = *j* − *i*. In other words, the predefined significance level is a function of length *L*_*i*, *j*_ of the detected parts in the segment. Here, let ${\widehat{T}}_{i,j}$ be the cut-off threshold of $\widehat{Z}$,

5 $$ \wp \left({\widehat{T}}_{i,j}\right)=\frac{\theta }{j-i} $$

with a given θ and a length that corresponds to a definite ${\widehat{T}}_{i,j}={\wp}^{-1}\left(\theta /\left(j-i\right)\right)$ based on using the inverse function of the cumulative distribution function. If $\widehat{Z}$ is less than ${\widehat{T}}_{i,j}$, then we will consider that there is no copy number change in the segment. Otherwise, it is necessary to split. The following is the criterion of segmentation in Eqn (6),

6 $$ \widehat{Z}=\underset{i,j}{\max}\left|\frac{\sum_{k=i}^{j-1}\left({x}_k-\widehat{\mu}\right)}{\widehat{\sigma}\sqrt{j-i}}\right|\ge {\widehat{T}}_{i,j} $$

When the constant parameter θ is subjectively determined, we define a new statistic *Z*_*i*, *j*_ by transforming formula (1) so that it represents a normalized standard deviation weighted by a predefined significance level between the two points *i* and *j*:

7 $$ {Z}_{i,j}=\frac{\sum_{k=i}^{j-1}\left({x}_k-\widehat{\mu}\right)}{{\widehat{T}}_{i,j}\sqrt{j-i}}={\omega}_{i,j}{\varepsilon}_{i,j} $$

where ${\omega}_{i,j}={\left({\widehat{T}}_{i,j}\sqrt{j-i}\right)}^{-1}$, *ω*_*i*, *j*_ > 0, and *ε*_*i*, *j*_ is the accumulated error between two points *i* and *j*, 1 < *i* < *j* < n + 1.

We select a point *p* between the start *1* and the end *n* in one segment. Thus, *Z*_1, *p*_ and *Z*_*p*, *n* + 1_ are the two statistics that correspond to the left side and the right side, respectively, of point *p* in the segment and represent the weighted deviation of these two parts. Furthermore, we define a new statistic *ℤ*_1, *n* + 1_(*p*),

8 $$ {\mathbb{Z}}_{1,n+1}(p)= dist\left(\left\{{Z}_{1,p},{Z}_{p,n+1}\right\},0\right) $$

where *dist*(〈∙〉, 0) is a distance measure between vector 〈∙〉 and 0. The Minkowski distance can be used here. These will be discussed in a later section “Selecting for the distance function”. Finally, we define a new test statistic *ℤ*_*p*_,

9 $$ {\mathbb{Z}}_p=\underset{1<p<n+1}{\max\ }{\mathbb{Z}}_{1,n+1}(p) $$

*ℤ*_*p*_ is the maximum of abrupt jumps of variance within the segment under the current constraints, and its position is found by iterating once over the whole segment. If *ℤ*_*p*_ is greater than the estimated standard deviation $\widehat{\sigma}$ at location *p*, that is, *ℤ*_*p*_ is considered significant, we will obtain a new candidate breakpoint *b* at *p*.

10 $$ b=\arg\ \underset{1<p<n+1}{\max\ }{\mathbb{Z}}_{1,n+1}(p) $$

Then, a binary segmentation procedure will be performed at breakpoint *b*, and we will apply the above algorithm recursively to the two segments *x*_1_, …, *x*_*p* − 1_ and *x*_*p*_, …, *x*_*n*_, *p* ∈ (1, *n*).

### Multi-scale scanning procedure

Up to now, the above algorithm has been able to identify the most significant breakpoints, except one short segment sandwiched between two long segments. In this case, the distance between breakpoints at the intermediate position *p* and both ends is much or far greater than 1. Thus, $\wp \left({\widehat{T}}_{1,p}\right)=\theta /p$ tends to 0, and ${\widehat{T}}_{1,p}$ has almost no change with an increase in *p*. The accumulated error generated by the sum process is equally shared to each point from 1 to *p*. When increasing the distance to the ends, the change of *Z*_*i*, *j*_ becomes slower. Thus, spike pulses and small segments embedded in long segments are suppressed. Therefore, if *ℤ*_*p*_ is less than the estimated standard deviation $\widehat{\sigma}$ after a one-time scan of the whole segment, we cannot arbitrarily exclude the presence of the breakpoint.

From the situation above, it is obvious that we cannot use the fixed endpoints to detect breakpoints on a global scale. This method is acceptable with large jumps or changes in long segments, but to detect shorter segments. We need smaller windows. For these smaller segments, scale-space scanning is used. In the DBS algorithm, in the second phase, a multi-scale scanning stage will be started by the windowed model, if a breakpoint was not found immediately by the first phase.

Here, let $\mathcal{W}$ be a width set of sliding windows, and a window width $\in \mathcal{W}$. Thus, the two statistics above, *Z*_1, *p*_ and *Z*_*p*, *n* + 1_, are updated to *Z*_*p* − *w*, *p*_ and *Z*_*p*, *p* + *w*_. The test statistic *ℤ*_*p*_ is updated by a double loop in Eqn (11),

11 $$ {\mathbb{Z}}_p=\underset{1<p<n,w\in \mathcal{W}}{\max\ } dist\left(\left\{{Z}_{p-w,p},{Z}_{p,p+w}\right\},0\right) $$

Therefore, we can find the local maximum across these scales (window width), which provides a list of (*Z*_*p* − *w*, *p*_, *Z*_*p*, *p* + *w*_, *p*, *w*) values and indicate that there is a potential breakpoint at *p* at the *w* scale. Once *ℤ*_*p*_ is greater than the estimated standard deviation $\widehat{\sigma}$, then a new candidate breakpoint is found. The new recursive procedure as the mentioned first phase will be applied to the two new segments just generated.

### Analysis of time complexity in DBS

In DBS, the first phase is a binary segmentation procedure, and the time complexity of this phase is O(*n* ∙ log *K*), where *K* is the number of segments in the result of the first phase, and *n* is the length of an observation sequence to be split. Because *n* ≫ *K*, the time complexity approaches O(*n*). Next, the second phase, the multi-scale scanning procedure, is costly compared with a one-time scan on a global scale on the whole segment. When $\mathcal{W}$ is a geometric sequence with a common ratio of 2, the time complexity of the second phase is O(*n* ∙ log *n*). When $\mathcal{W}$ includes all integer numbers from *1* to *n*, the time complexity of the second phase degenerates to O(*n*^2^). Then, in this case, the algorithm framework of DBS is fully equivalent to one in BACOM, which is similar to the idea used in Circular Binary Segmentation procedure.

In simulation data set, it is not common that one short segment sandwiched between two long segments is found in the first or first few dichotomies of whole segmentation process, because broader changes can be expected to be detected reasonably well. After several recursive splits were executed, the length of each sub-segment is greatly reduced. Then, the execution time of the second phase in DBS is also greatly reduced at each sub-segment. But the second phase must be triggered once before the recursive procedure ends. So, the time complexity of DBS tends to approach O(*n* ∙ log *n*). Moreover, the real data is more complicated, so the effect of DBS is O(*n* ∙ log *n*) in practice. Its time complexity is about the same as its predecessors, but DBS is faster than them. We will discuss later in section “Computational Performance”.

### Convergence threshold: Trimmed first-order difference variance $\widehat{\boldsymbol{\sigma}}$

Here, the average of estimated standard deviation $\widehat{\sigma}$ on each chromosome is the key to the convergence of iterative binary segmentation, and it comes from a trimmed first-order difference variance estimator [19]. Combined with simple heuristics, this method may be used to further enhance the accuracy of $\widehat{\sigma}$. Suppose we restrict our attention to exclude a set of potential breakpoints by computationally inexpensive methods. One way to identify potential breakpoints is to use high-pass filters, i.e., a filter obtaining high absolute values when passing over a breakpoint. The simplest such filter uses the difference *∆x*_*i*_ = *x*_*i* + 1_ − *x*_*i*_, 1 < *i* < *n* for each position *i*. We calculate all the differences at each position and identify approximately 2% of the probe positions as potential breakpoints. In other words, the area below the 1st percentile and above the 99th percentile of all differences corresponds to the breakpoints. Then, we estimated the standard deviation ${\overset{\sim }{\sigma}}^{\prime }$ of *∆x*_*i*_ at the remaining positions. Supposing the change of the variances of each segment on one chromosome is not very large, the average standard deviation $\widehat{\sigma}$ of each segment is $\widehat{\sigma}={\overset{\sim }{\sigma}}^{\prime }/\sqrt{2}$.

We need to be reminded that the current $\widehat{\sigma}$ is only used to determine whether to continue to split iteratively. After a whole binary segmentation procedure is completed, we can obtain preliminary results and a corresponding binary tree of the test statistic *ℤ*_*p*_ generated by segmentation. Furthermore, according to the binary tree, a new fine-tuned ${\widehat{\sigma}}^{\prime }$ will be generated naturally to improve the intra-segment variance more accurately. Finally, we select those candidate breakpoints in which *ℤ*_*p*_ is greater than the given ${\widehat{\sigma}}^{\prime }$ as the final ‘true’ breakpoints.

### Determining real breakpoints or false breakpoints

**Fig. 1:**
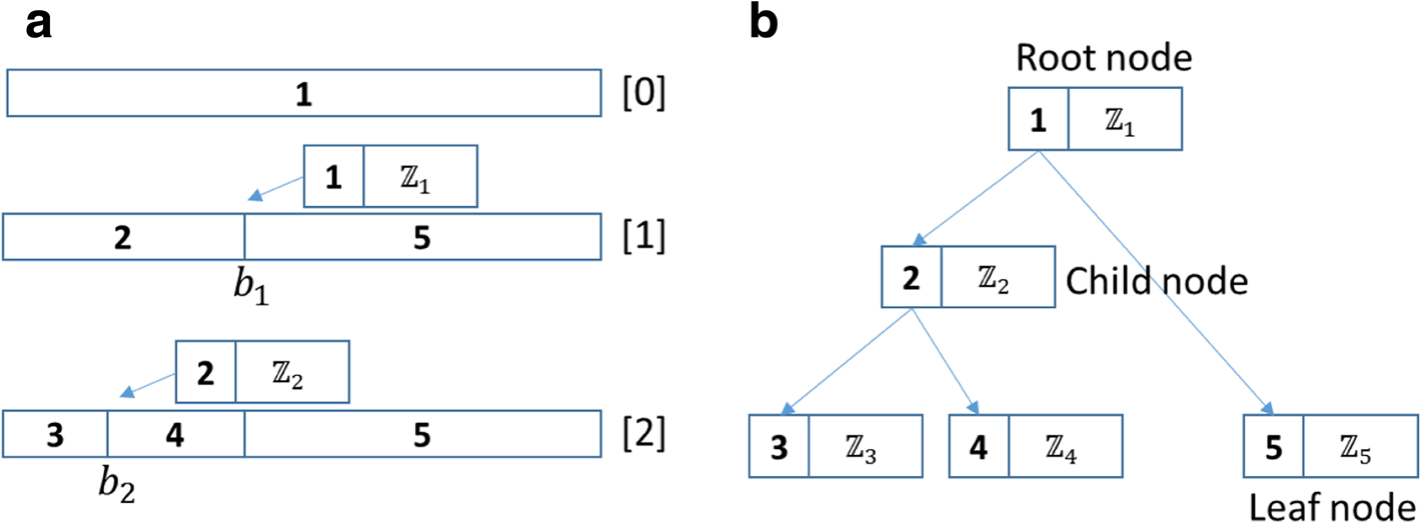
Segmentation process and binary tree of *ℤ*_*p*_ in DBS. **a** an assumed segmentation process with two breakpoints. Row [0] is the initial sequence to be split. Row [1] shows the first breakpoint would be found at loci *b*_1_, and Row [2] is similar. **b** shows the corresponding binary tree of *ℤ*_*p*_ generated by (**a**). Here the identification of every node (Node ID) also is the Segment ID

Let us now analyze the specific process of ${\widehat{\sigma}}^{\prime }$ in detail. Figure 1(a) shows an assumed complete segmentation process. After being split twice at breakpoints *b*_1_ and *b*_2_, an initial array (Segment ID is 1) is divided into three segments (their IDs are 3, 4, and 5). *ℤ*_1_ and *ℤ*_2_ are two local maximums *ℤ*_*p*_ at the corresponding breakpoints of two segments (IDs are 1 and 2). If *ℤ*_3_, *ℤ*_4_ and *ℤ*_5_ within the corresponding segments are all less than the pre-calculated $\widehat{\sigma}$, then the whole subdivision process ends.

Then, we can generate a corresponding binary tree of test statistic *ℤ*_*p*_; see Fig. 1(b). The values of the root node and child nodes are *ℤ*_*p*_ of the initial array and the corresponding intermediate results, and the values of the leaf nodes are *ℤ*_*p*_ of the results of segmentation. The identification of every node (Node ID) is the Segment ID.

We define a new distance η between the set of non-leaf nodes and the set *N*_*leaf*_ of leaf nodes,

12 $$ \upeta =\min \left({\mathbb{Z}}_i|i\notin {N}_{\mathrm{leaf}}\right)-\max \left(\widehat{\sigma_j}|j\in {N}_{\mathrm{leaf}}\right) $$

where *ℤ*_*i*_ is the *ℤ*_*p*_ of corresponding segments, and $\widehat{\sigma_j}$ is the estimated standard deviation of corresponding segments. Because now the partitions have been initially completed, we use the real local standard deviation of each segment to examine the significance level of every child node.

If η > 0, all *ℤ*_*p*_s of non-leaf nodes are greater than all standard deviations of leaf nodes, and the breakpoints corresponding to all non-leaf nodes are the real significant breakpoints and the DBS algorithm ends immediately.

If η ≤ 0, there are false breakpoints resulting in over-segmentation, which are less than the standard deviation of the leaf nodes. Thus, we update $\widehat{\sigma}$ to ${\widehat{\sigma}}^{\prime }$,

13 $$ {\widehat{\sigma}}^{\prime }=\max \left(\widehat{\sigma_j}|j\in {N}_{\mathrm{leaf}}\right)+\lambda $$

where *λ* is a safe distance between the non-leaf nodes and the leaf nodes. Its default value is 0.02. In other words, we only choose the candidate breakpoints whose *ℤ*_*p*_ are greater than ${\widehat{\sigma}}^{\prime }$ as the final result. Here when a false breakpoint is removed, then the sub-segments corresponding to its two children are merged. This pruning process is equivalent to the process of merging and collating segments in other algorithms. In the following sections, we will discuss segmentation process using simulation data and actual data sample.

Here it needs to be emphasized that the proper over-segmentation is helpful to avoid missing real breakpoints possibly exist. In actual data, if and only if η trends closer to zero, the best segmentation result will be obtained due to the continuity of variance within segments. We will discuss later in section “Segmentation of actual data sample”.

- Quickly calculating the statistic *Z*_*ij*_

In DBS, we use absolute errors rather than squared errors to enhance the computational speed of segmentation. The time complexity of absolute errors can be reduced to O(1), and it only needs one subtraction operation for the summing of one continuous region using an integral array. The algorithm of integral array is naturally decreased from the integral image in computing 2D images [20]. A special data structure and algorithm, namely, summed area array, make it very quick and efficient to generate the sum of values in a continuous subset of an array.

Here, we only need to use a one-dimensional summed area table. As the name suggests, the value at any point *i* in the summed area array is just the sum of all the left values of point *i*, inclusive: ${\mathrm{S}}_i={\sum}_{k=1}^i{x}_k$. Moreover, the summed area array can be computed efficiently in a single pass over a chromosome. Once the summed area array has been computed, the task of evaluating the sum between point *i* and point *j* requires only two array references. This method allows for a constant calculation time that is independent of the length of the subarray. Thus, using this fact, the statistic *Z*_*ij*_ can be computed rapidly and efficiently in Eqn (14).

14 $$ {Z}_{i,j}={\omega}_{i,j}{\sum}_{k=i}^{j-1}\left({x}_k-\widehat{\mu}\right)={\omega}_{i,j}\left[{\mathrm{S}}_j-{\mathrm{S}}_i-\left(j-i\right)\widehat{\mu}\right] $$

where ${\omega}_{i,j}={\left({\wp}^{-1}\left(\theta /\left(j-i\right)\right)\sqrt{j-i}\right)}^{-1}$ and ℘^−1^(∙) are the inverse functions of the cumulative distribution function of *N* (0, 1).

- Selecting for the distance function *dist*(∙)

The two statistics *Z*_1, *p*_ and *Z*_*p*, *n* + 1_ are weighted standard deviations between point *p* and two ends, respectively,

15 $$ {Z}_{1,p}={\omega}_{1,p}{\varepsilon}_{1,p} $$

16 $$ {Z}_{p,n+1}={\omega}_{p,n+1}{\varepsilon}_{p,n+1} $$

Because ${\varepsilon}_{i,j}={\sum}_{k=i}^{j-1}\left({x}_k-\widehat{\mu}\right)$, then *ε*_1, *p*_ + *ε*_*p*, *n* + 1_ = 0. Thus,

17 $$ {\mathbb{Z}}_{1,n+1}(p)= dist\left(\left\langle {Z}_{1,p},{Z}_{p,\mathrm{n}+1}\right\rangle, 0\right)= dist\left(\left\langle {\omega}_{1,p},{\omega}_{p,n+1}\right\rangle, 0\right)\left|{\varepsilon}_{1,p}\right|={\mathfrak{D}}_p\left|{\varepsilon}_{1,p}\right| $$

Finally, *ℤ*_1, *n* + 1_(*p*) represents an accumulated error |*ε*_1, *p*_| weighted by ${\mathfrak{D}}_p$. The test statistic *ℤ*_*p*_ physically represents the largest fluctuation of *ℤ*_1, *n* + 1_(*p*) on a segment.

There are two steps in the entire process of searching for the local maximum *ℤ*_*p*_ on a segment*.* First, the Minkowski distance (*k* = 0.5) is used to find the position of breakpoint *b* at the local maximum by the model Eqn (18).

18 $$ {\mathfrak{D}}_p={dist}_{k=0.5}\left(\left\langle {\omega}_{1,p},{\omega}_{p,n+1}\right\rangle, 0\right)={\left({\omega_{1,p}}^k+{\omega_{p,n+1}}^k\right)}^{1/k} $$

When *k* < 1, the Minkowski distance between 0 and 〈*ω*_1, *p*_, *ω*_*p*, *n* + 1_〉 tends to the smaller component within *ω*_1, *p*_ and *ω*_*p*, *n* + 1_. Furthermore, *ω*_1, *p*_ and *ω*_*p*, *n* + 1_ belong to the same interval [*ω*_1, *n* + 1_, *ω*_1, 2_], and as *p* moves, they exhibit the opposite direction of change. Thus, when *ω*_1, *p*_ is equal to *ω*_*p*, *n* + 1_, ${\mathfrak{D}}_p$ reaches a maximum value, and then *p* = *n*/2.

From the analysis above, when *p* is close to any end (such as *p* is relatively small, then *n* − *p* is sufficiently large), *Z*_1, *p*_ is very susceptible to the outliers between point *1* and *p*. In this case, the position of such a local maximum *Z*_1, *p*_ may be false breakpoints, but *Z*_*p*, *n* + 1_ is not significant because there are a sufficient number of probes between point *p* and another end to suppress the noise. Here, the Minkowski distance (*k* < 1) is used to filter these unbalanced situations. At the same time, the breakpoints at balanced local extrema are preferentially found. Usually, the most significant breakpoint may be found at the middle of a segment due to ${\mathfrak{D}}_p$, so the performance and stability of binary segmentation is increased.

Once a breakpoint at *b* is identified, *ℤ*_1, *n* + 1_(*b*) can be calculated by the Minkowski distance (*k* ≥ 1). The Minkowski distance (*k* < 1) is not a metric, since this violates the triangle inequality. Here, we select the Chebyshev distance by default:

19 $$ {\mathbb{Z}}_p={\mathbb{Z}}_{1,n+1}(b)=\max\ \left(\left|{Z}_{1,b}\right|,\left|{Z}_{b,n+1}\right|\right) $$

In other words, at each point, *ℤ*_1, *n* + 1_(*p*) tends to the smaller value of the left and right parts to suppress the noise when searching for breakpoints; *ℤ*_1, *n* + 1_(*p*) tends to the larger value for measuring the significance of breakpoints.

### Algorithm DBS: Deviation binary segmentation

Input: Copy numbers *x*_1_, …, *x*_*n*_; predefined significance level θ = 0.05; filtration ratio γ = 2(%); safe gap λ = 0.02;

Output: Indices of breakpoints *b*_1_, …, *b*_*K*_; segment average *y*_1_, …, *y*_*K*_; and degree of significance of breakpoints ${\mathbb{Z}}_{b_1},\dots, {\mathbb{Z}}_{b_k}$.

1. Calculate integral array by letting *S*_0_ = 0, and iterate for *i* = 1…*n*:

$$ {\mathrm{S}}_i={\mathrm{S}}_{i-1}+{x}_i $$

2. Estimate standard deviation $\widehat{\sigma}$:
  - Calculate the differences iteratively for *i* = 1…*n*: *d*_*i*_ = *x*_*i* + 1_ − *x*_*i*_
  - Sort all *d*_*i*_ and exclude the area below the γ/2 percentile and above the 100 − γ/2 percentile of differences of *d*_*i*_, then calculate the estimated standard deviation ${\overset{\sim }{\sigma}}^{\prime }$ at the remaining part.
  - Get $$ \widehat{\sigma}={\overset{\sim }{\sigma}}^{\prime }/\sqrt{2} $$.
3. Start binary segmentation with two fixed endpoints for segment *x*_1_, …, *x*_*n*_, and calculate the average $\widehat{\mu}$ on the segment;
  - By Eqn (14) iterate *Z*_1, *p*_ and *Z*_*p*, *n* + 1_ for *p* = 1…*n*;

then get *ℤ*_1, *n* + 1_(*p*) by Eqn (18), and *k* = 0.5;

b) Search the index of potential breakpoint *b*_*k*_ at which *ℤ*_*p*_ is the maximum in the previous step, and calculate ${\mathbb{Z}}_{b_k}$ by Eqn (19);
c) If ${\mathbb{Z}}_{b_k}>\widehat{\sigma}$, store ${\mathbb{Z}}_{b_k}$ and *b*_*k*_, then go to Step 3 and apply binary segmentation recursively to the two sub-segments ${x}_1,\dots, {x}_{b_k-1}$ and ${x}_{b_k},\dots, {x}_n$; otherwise, the multi-scale scanning will be started, and enter Step 4.

4. Start binary segmentation with various sliding windows for segment *x*_1_, …, *x*_*n*_,
  g) Create a width set of sliding windows by letting ${\mathcal{W}}_0=n/2$, and iterate ${\mathcal{W}}_i={\mathcal{W}}_{i-1}$/2 until ${\mathcal{W}}_i$ is less than 2 or a given value.
  h) Similar to the above binary segmentation, iterate *Z*_*p* − *w*, *p*_, *Z*_*p*, *p* + *w*_ and *ℤ*_1, *n* + 1_(*p*) for *p* = 1…*n* under all sliding windows ${\mathcal{W}}_i$, then find the index of potential breakpoint *b*_*k*_ by the maximum and ${\mathbb{Z}}_{b_k}$ is calculated.
  i) If ${\mathbb{Z}}_{b_k}>\widehat{\sigma}$, store ${\mathbb{Z}}_{b_k}$ and *b*_*k*_, then go to Step 3 and recursively start a binary segmentation without windows to the two segments ${x}_1,\dots, {x}_{b_k-1}$ and ${x}_{b_k},\dots, {x}_n$; otherwise, terminate the recursion and return.
5. Merge operations: calculate η and update ${\widehat{\sigma}}^{\prime }$, and prune child nodes corresponding to candidate breakpoints to satisfy η > λ.
6. Sort the indices of breakpoints *b*_1_, …, *b*_*K*_, find the segment averages:

*y*_*i*_ = average(${x}_{b_{i-1}},\dots, {x}_{b_i-1}$) for *i* = 1…*K*, and *b*_0_ = 1.

In the algorithm, we use the data from each segment to estimate the standard deviation of noise. As it is well documented that the copy number signals have higher variation for increasing deviation from diploid ground states, by assuming each segment has the same copy number state, the segment-specific estimate of noise level makes the algorithm robust to the heterogeneous noise.

DBS is a statistical approach based on observations. Similar to its predecessors, there is a limit of minimum length of short segments in order to meet the conditions of CLT. In DBS, the algorithm has also been extended to allow a constraint on the least number of probes in a segment. It is worth noting that low density data, such as arrayCGH, is not suitable for DBS, and an insufficiently low limit on the length (twenty probes) of a segment is necessary.

## Results and discussion

### Constructing the binary tree of *ℤ*_***p***_ and filtering the candidate breakpoints

In DBS, the selection of parameters is not difficult. There are three parameters. The predefined significance level θ can be seen as a constant. The filtration ratio γ implies the proportion of breakpoints at one original sequence. When breakpoints are scarce in copy number analysis, the 2% rejection rate is already sufficient. The safety gap λ should be a positive number close to zero in determining the trade-off between high sensitivity and high robustness (i.e., a leap at the breakpoint visually). It limits the minimum of significant degrees *ℤ*_*p*_ of breakpoints in the results and ensures that the most significant breakpoints are not intermixed with some inconspicuous breakpoints.

The key factor is the average of estimated standard deviation $\widehat{\sigma}$ of all segments and is predicted statistically according to the difference between adjacent points. When the preliminary segmentation is completed, $\widehat{\sigma}$ will also be updated in terms of the binary tree of *ℤ*_*p*_. Therefore, the binary tree generated by DBS is the key to the judgment of breakpoints.

**Fig. 2:**
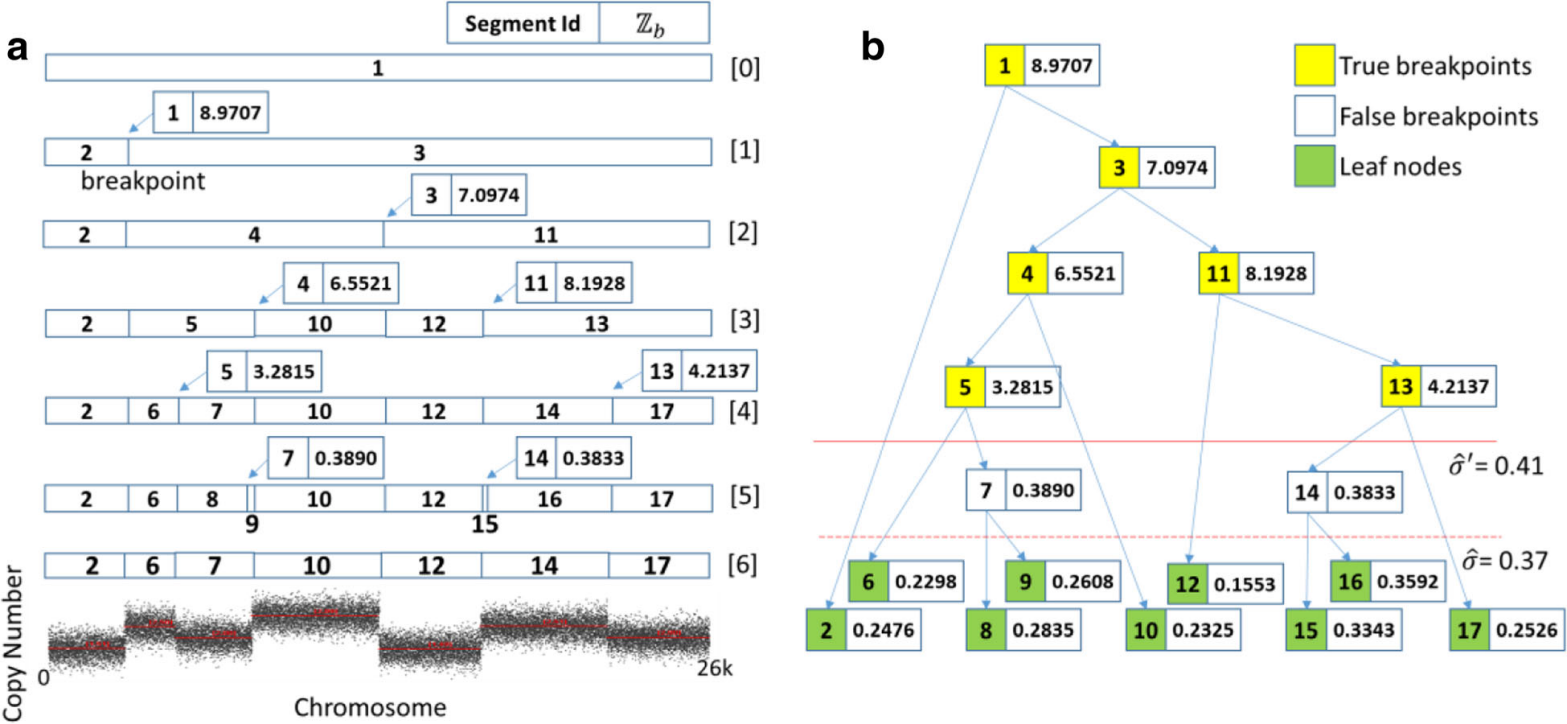
Segmentation process with simulation data in DBS. **a** shows the segmentation process by splitting multiple times. Notably, DBS uses a recursive algorithm. After Node 1, 3, 4, 5, and 7 were found one by one, Node 11, etc. at right part were discovered. The red lines over gray data points is the segmentation curves. The curves are the results of segmentation, and indicate the ranges and average of each sub-segment. **b** shows the corresponding binary tree of *ℤ*_*p*_ generated by the left panel (**a**). The red dotted line represents the position of the estimated standard deviation $\widehat{\sigma}$, and the red solid line represents the position of the threshold ${\widehat{\sigma}}^{\prime }$ of degree of significant *ℤ*_*p*_ of breakpoints

Consider first the simple condition to segment simulation data generated by random numbers that follows several normal distributions. Here, Fig. 2 demonstrates a segmentation process using DBS. In Fig. 2(a), $\widehat{\sigma}$ is 0.3703 calculated by DBS but is less than the actual value 0.4 due to the strong filtering effect of γ. Two percent of the length of simulation data is much larger than the actual six breakpoints. After splitting several times, the initial array in the zero row is divided into nine segments in the fifth row. Now, *ℤ*_*p*_ of the result segments is less than the predetermined threshold $\widehat{\sigma}$, then the whole subdivision process ends, and the binary tree of *ℤ*_*p*_ is generated in Fig. 2(b). Next, we estimate the maximum standard deviation of the nine segments, and it is close to 0.4. Thus, η = 0.3833 − 0.4 < 0, and $\widehat{\sigma}$ is updated from 0.37 to 0.41 at least. Then, in Fig. 2(b), Node 7 and Node 14 are classified as two false breakpoints, so their children are pruned and they degenerate into leaf nodes. Thus, only seven segments are split in the sixth row because there are six yellow candidate breakpoints whose *ℤ*_*p*_ are greater than ${\widehat{\sigma}}^{\prime }$.

### Segmentation of actual data sample

**Fig. 3:**
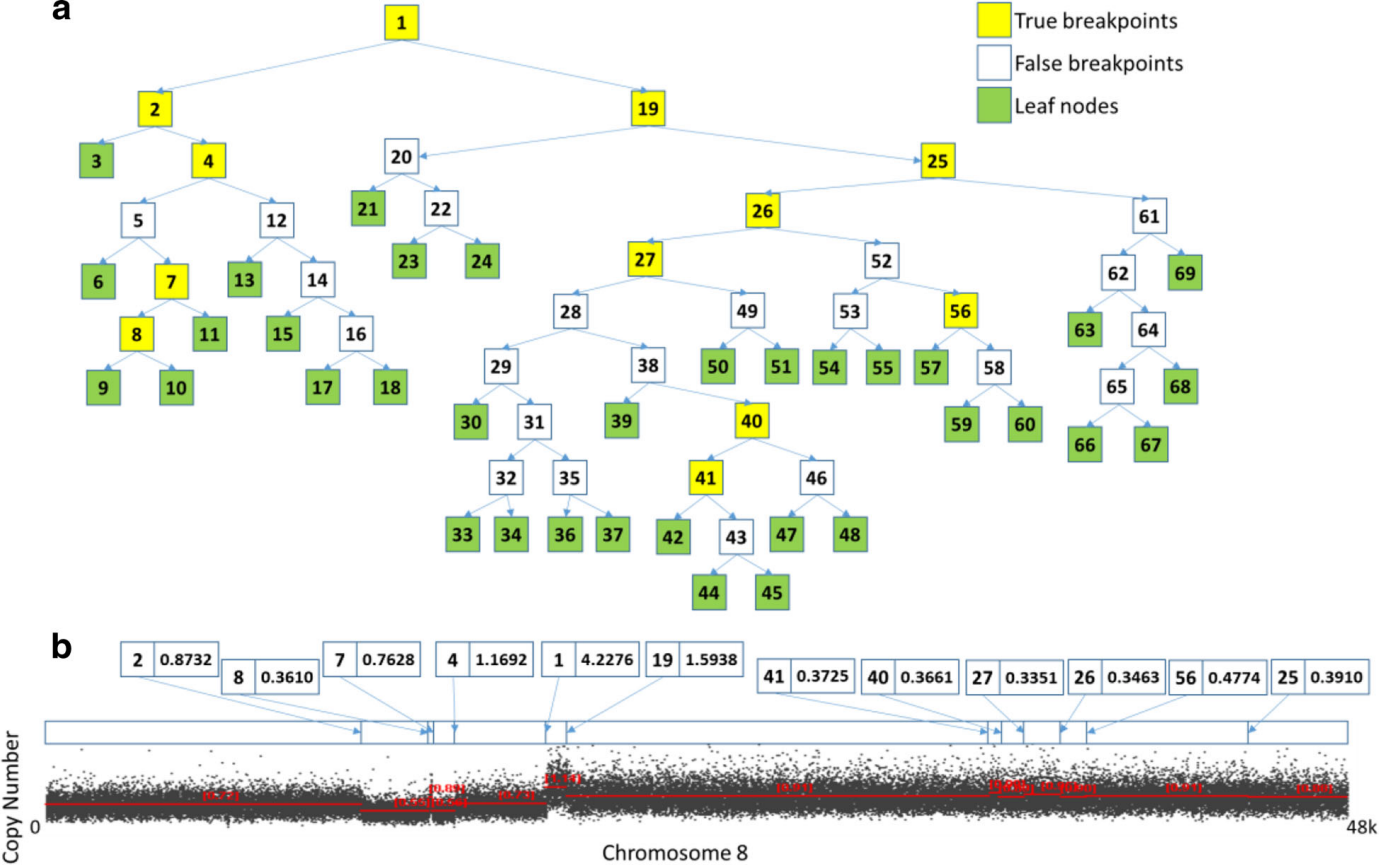
Segmentation process with an actual data sample in DBS (using half copy numbers). **a** the segmentation process in the binary tree of *ℤ*_*p*_. **b** plots the copy number of an actual sample, and shows the position and *ℤ*_*p*_ of the 12 true breakpoints, which correspond to these yellow nodes in Panel (**a**). In (**b**), the observed copy number signals are the ratios of the measured intensity of tumor-normal matched sample

Now, we illustrate splitting one actual data sample using copy numbers. One pair of tumor-normal matched samples is picked from the TCGA ovarian cancer dataset (TCGA_OV), with the sample ID named TCGA-24-1930-01A-01D-0648-01. In Fig. 3, we chose chromosome 8 as the focus of analysis, which contains different lengths of segments, especially one short segment sandwiched between two long segments (resembles a sharp pulse). In this example, $\widehat{\sigma}$ is estimated to be 0.2150 by the default parameters in DBS. Then, the initial array is divided into 35 segments (leaf nodes) by the separations recursively in Fig. 3(a). Next, we estimate the maximum standard deviation of the segments corresponding to these leaf nodes, and the maximum is 0.3121. However, the minimum *ℤ*_*p*_ of the non-leaf nodes is 0.2197, so η < 0, and $\widehat{\sigma}$ is updated to approximately 0.33. Then, as shown in Fig. 3(a), the 22 white nodes are classified as false breakpoints whose *ℤ*_*p*_ is less than 0.33. Finally, the result includes 12 true breakpoints corresponding to these yellow nodes. Figure 3(b) shows the position and the degree of significance of the *ℤ*_*p*_s of all true breakpoints. We can see that the most significant change is found at the location of Node 1 in the first scan, and its *ℤ*_*p*_ is also the maximum significant degree (4.2276) of all breakpoints. Next, the more significant Node 2, Node 4 and Node 19 are found one by one, and they occupy four-fifths of the top 5 significant breakpoints. This result is consistent with the visual appearance in Fig. 3(b).

However, the last of the top 5 significant breakpoints was not immediately found. Here, we can see that the *ℤ*_*p*_ of Node 4 is rising instead of falling compared to that of its parent Node 2. This fact also predicts the existence of complex changes between Node 2 and its child, Node 4. The resolution capacity of the binary segmentation with two fixed endpoints in DBS is increased as the length of the split segments becomes shorter, unless the binary segmentation with various sliding windows is triggered. After splitting several times, one sharp pulse is found between Node 7 and Node 8. The processes of discovering Node 40, Node 41 and Node 56 are also similar.

In DBS, because the resolution capacity of breakpoints is continually enhanced with recursive segmentation, the binary segmentation with multi-scale scanning will be performed at the leaf nodes. Thus, the segments corresponding to the leaf nodes cannot contain the breakpoints whose *ℤ*_*p*_ is greater than $\widehat{\sigma}$. Therefore, the conditional multi-scale scanning of DBS determining the trade-off between segmentation efficiency and segmentation priority (i.e., the sooner the greater) can be accepted, although this method leads to the destruction of the tree structure. There will be no missing breakpoints; this process will merely postpone the time to find them unless $\widehat{\sigma}$ is overestimated.

Usually, the segmentation process of Node 19 is representative, as the *ℤ*_*p*_ of child nodes are monotonically decreasing. The possibility of finding breakpoints is smaller after shortening the segment length and excluding more significant breakpoints continuously. The *ℤ*_*p*_ of new nodes will eventually be less than the $\widehat{\sigma}$ predicted previously by CLT, and a new leaf node will be identified to terminate the recursion.

For the above examples, we argue that the proper underestimation of $\widehat{\sigma}$ is necessary. It ensures that the leaf nodes cannot contain any real breakpoints in the initial top-down segmentation process. Simultaneously, the standard deviations of the segments corresponding to the leaf nodes also correctly reflect the actual dispersion under correct segmentation, which guides the classification by degree of significance of breakpoints through a bottom-up approach. In DBS, we choose a sufficiently large filtration ratio γ to ensure this result. Thus, η would be less than 0 after the preliminary segmentation. Otherwise, there is a reason to worry about missing breakpoints, which can be observed only when the change in adjacent segments is more obvious, as shown in Fig. 2.

### Test dataset

To generate a test dataset that has a similar data structure with that in real cases, we chose real data samples as the ground truth reference [16]. We manually checked the plots of all chromosomes and chose several genomic regions as the reference templates for generating simulated data. These regions are representative of the diversity of copy number states that are typically extracted in tumor-normal sample pairs by classical segmentation algorithms and have no structural variations in them. In addition, the data included in each template follows a single Gaussian distribution, and there are four different templates corresponding to copy number range from 1 to 4. Using these templates, we generate a test dataset at the assured position of breakpoints and the given average copy number for each segment.

Furthermore, since the templates have been normalized, they can be viewed as pure cancer samples. We can generate simulated copy number profiles with any proportion of normal cells contaminated. Here, we chose several different proportions between 30 and 70%. For one region of length *n* in the test data, *n* data points are sampled from the template, which corresponds to the appointed average copy number. Then, according to model (6), *x*_*i*_ is transferred from sampling data *p*_*i*_ in the template with normal cell contamination, and *α* is the fraction of normal cell subpopulation in the sample*.*

20 $$ {x}_i={p}_i\ast \left(1-\alpha \right)+2\ast \alpha $$

The entire test dataset consists of 104 test sequences and a total of 876 test segments. The length of each detected sequence is between about 10^3^ and 10^5^. In the process of calculation, too short sequences are too vulnerable to external random interference, which is generated by other programs running in operating system. Therefore, we only use sequences of length more than 10,000 in performance analysis.

### Test method

We evaluate the performance of these algorithms by calculating the precision of segmentation results. With reference to test methods proposed by the pioneers [12, 21], a classification was constructed to test and compare the sensitivity and specificity of segmentation algorithms, which are used for the detection of recurring alterations in Multiplex Ligation-dependent Probe Amplification (MLPA) analysis [12].

A binary classifier with a parameter τ>0 was proposed using aberration calling. Its τ is a discrimination threshold, which determines the sensitivity of the aberration calling. The classifier outcome is a discrete class label, indicating that the point to be tested is in the normal region or aberrant region. Then, Eqn (21) is given, where *p* is a location to be detected, and *μ*_*k*_ is the average of copy number of the segment Seg_*k*_ including *p*. (The expected DNA copy number in normal cells is two.)

21 $$ \mathrm{Tag}(p)=\left\{\left|{\mu}_k-2\right|<\uptau, p\in {\mathrm{Seg}}_k\right\} $$

If Tag(*p*) is true, we consider that the *p* is in the normal region and is called positive. Otherwise, it is in an aberrant region and is called negative. When we use the given breakpoints and the average copy number of segments in the test dataset, the gold standard was obtained by uniform sampling near the given breakpoints. In the gold standard, there were 1424 positive and 4752 negative values used for the comparison.

Thus, the test mechanism that was independent of the structure of the algorithm was established for segmentation accuracy. If the prediction from the classifier using the outcomes of a segmentation algorithm is positive and the actual value in the gold standard is also positive (normal loci), then it is called a true positive (TP); however, if the actual value is negative (aberrant loci), then it is said to be a false positive (FP). Conversely, a true negative (TN) has occurred when both the prediction outcome and the actual value are negative, and a false negative (FN) is when the prediction outcome is negative, while the actual value is positive.

### Segmentation accuracy

**Fig. 4:**
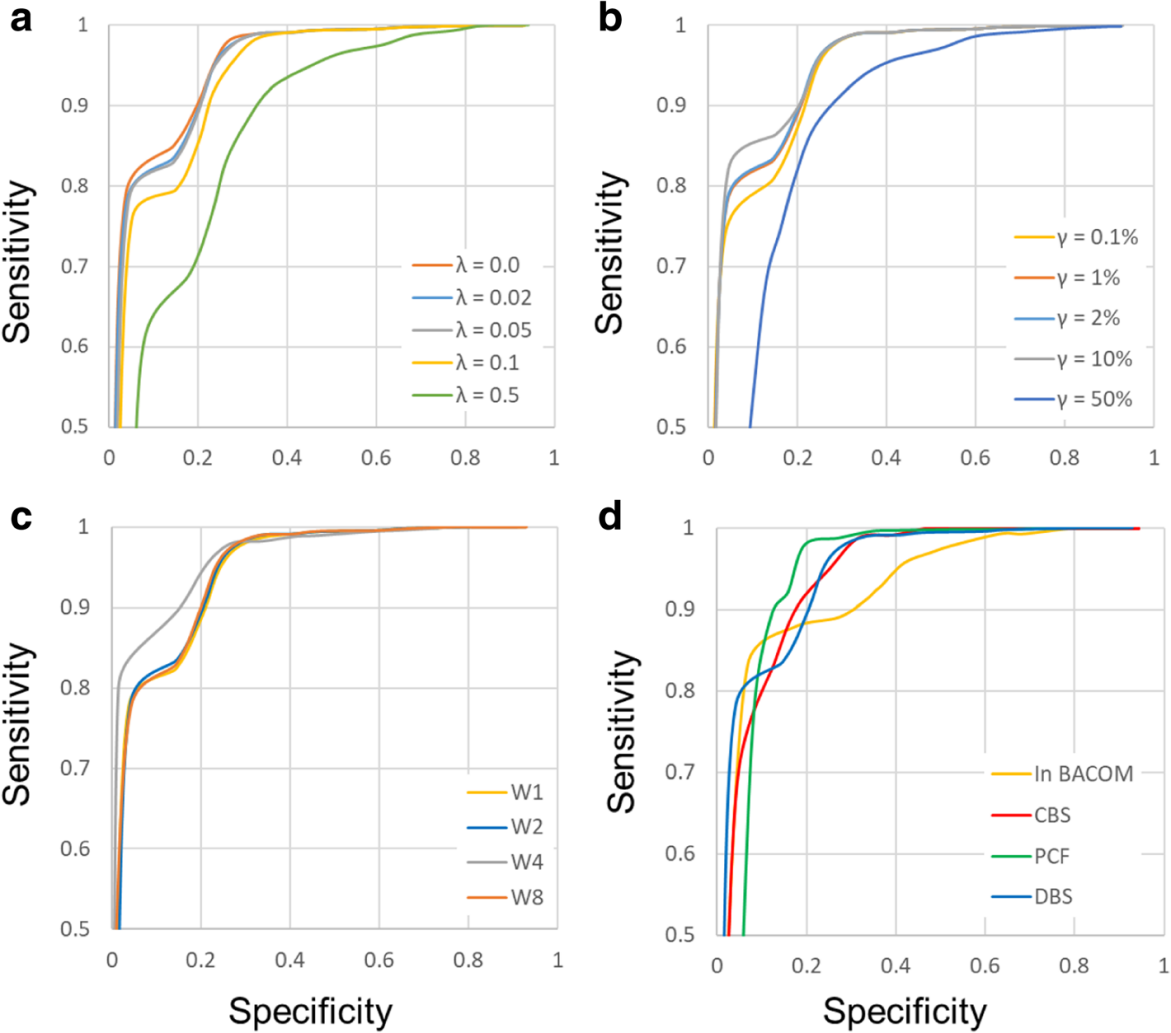
ROC-curves of five segmentation methods. The curves show the sensitivity and specificity of accuracy for a sequence of thresholds as calculated by comparing aberration calls to the classifications made in a MLPA-analysis on the test dataset. (**a**) and (**b**) show that the classification accuracy is not affected much for a wide range of λ and γ. Here γ is equal to 0.02 in (**a**), and λ is equal to 0.02 in (**b**). **c** shows the effect of different combinations of window sizes. Curve W1 is the result using window sizes generated by the arithmetic progression with common difference of 1. Curve W2, W4 and W8 correspond to window sizes of the geometric sequence with common ratio of 2, 4 and 8 respectively. λ and γ is default value (0.02). **d** shows calls based on the segmentations found by DNAcopy v1.52.0 (CBS), copynumber v1.18 (PCF), the method in BACOM and DBS with raw data

Using the MLPA binary classifier as the gold standard, the sensitivity and specificity of aberration calling were calculated for a range of threshold values θ. Figure 4 shows the resulting Receiver Operating Characteristic (ROC) curves, and panel (a) and (b) illustrate the results for DBS depending on the choices of λ and γ. Because these two parameters have actual physical meaning and the value ranges are bounded, aberration calling appears to be almost independent of the parameters by rational choice.

Panel (c) shows the effect of different combinations of window sizes. Curve W1 is the result using window sizes generated by the arithmetic progression with common difference of 1, and corresponds to use arbitrary sets of window sizes. Curve W2, W4 and W8 correspond to window sizes of the geometric sequence with common ratio of 2, 4 and 8 respectively. We can see that different combinations have little effect on the segmentation result.

Panel (d) shows calls made on the basis of the segmentations found by DBS, PCF, Circular Binary Segmentation (CBS) [3] and the segmentation method in BACOM [15, 16] with raw data. In comparison studies of the accuracy of the segmentation solutions, CBS is the most commonly used available algorithm and has good performance in terms of sensitivity and false discovery rate. PCF is a relatively new copy number segmentation algorithm based on least squares principles and combined with a suitable penalization scheme. Here the recent versions have been used with the original R implementations, DNAcopy v1.52.0 (CBS) and copynumber v1.18 (PCF). The predecessor of DBS is the segmentation method in BACOM, and this precursor replaces a decision process-based permutation test on CBS with a decision process based on Central Limit Theorem (CLT). There are the differences between DBS and CBS mainly in the following points. Firstly, the criterion of segmentation use Eqn (6) in BACOM, however Eqn (9) is the criterion in DBS. Secondly, the algorithm structure of the method in BACOM mainly contains a complete double circulation with recursively dividing into three sub-segments. DBS only contains a single circulation with recursive splits. Finally, the test statistics of the method in BACOM and the first phase in DBS are calculated point by point, this is equivalent to a scan process using window sizes with the arithmetic progression with common difference of 1. But the sliding windows of the second phase in DBS are a geometric sequence with a common ratio of 2.

In terms of aberration calling accuracy, Table 1 shows that CBS, PCF, DBS and others give nearly similar good results using the default parameter settings. The AUC of the ROC curves (in Fig. 4 (d)) corresponding to the four algorithms all exceed 0.9. In other words, all algorithms have detected most of the real breakpoints. However, ROC curve cannot detect whether over-segmentation exist. Because the average of sub-segment separated further based on correct segmentation will not change.

**Table 1:** Segmentation and Merging effects

	AUC	Segment Count	Over-segmentation Ratio
In BACOM	0.9256	845	0.965
CBS	0.9373	1171	1.34
PCF	0.9279	4906	5.60
DBS	0.9452	967	1.104

Here the entire test dataset consists of 104 test sequences and a total of 876 test segments. The AUC correspond to the ROC curves in Fig. 4(d). Segment count is the number of segments generated by the four algorithms. Over-segmentation ratio is a ratio of the segment count generated by each algorithm and the actual number of test segments

We found that the number of segments included in the segmentation result is different. The entire test dataset consists of 104 test sequences and a total of 876 test segments. The method in BACOM only got 845 segments, it shows the existence of under-segmentation. There is more serious over-segmentation in PCF using default parameter (it is too small). In CBS and DBS, the results are all over-segmented. But the result of DBS is slightly better than CBS. Here we defined a variable, over-segmentation ratio, which is a ratio of the segment count generated by each algorithm and the actual number of test segments. As shown in Table 1, the ratio of DBS is the minimum in all over-segmentation results. It also shows the merit of the merge operation (pruning false breakpoints) in DBS.

### Computational performance

We further compare the computational performance of the segmentation solutions found by the four above-mentioned methods. We tested the entire test dataset with four algorithms and collected the computation time (in seconds) per sample. Using default parameter settings, we compared the computing times of DBS, CBS, PCF and the method in BACOM on the 10 samples in the 26 K simulation data set, on 10 samples in 160 K simulation data set, and on 10 samples from Affymetrix SNP 6.0 Array. Table 2 gives the average computation time (in seconds) per sample. With default preprocessing of the data, on average, DBS is about 5 times faster than PCF, is about 15 times than CBS, and is about 23 times than the method in BACOM.

**Table 2:** Computational performance

Method	Time (s)			Trend Line	
	Test 1 (26 K, 1 Chr.)	Test 2 (160 K, 1 Chr.)	Affymetrix SNP 6.0 (868 K, 22 Chr.)	Slope	R^2^
In BACOM	0.592	18.769	34.818	1.999	0.988
CBS	1.442	6.249	80.551	0.984	0.963
PCF	0.393	2.638	10.256	0.968	0.979
DBS	0.093	0.524	2.167	0.968	0.986

**Fig. 5:**
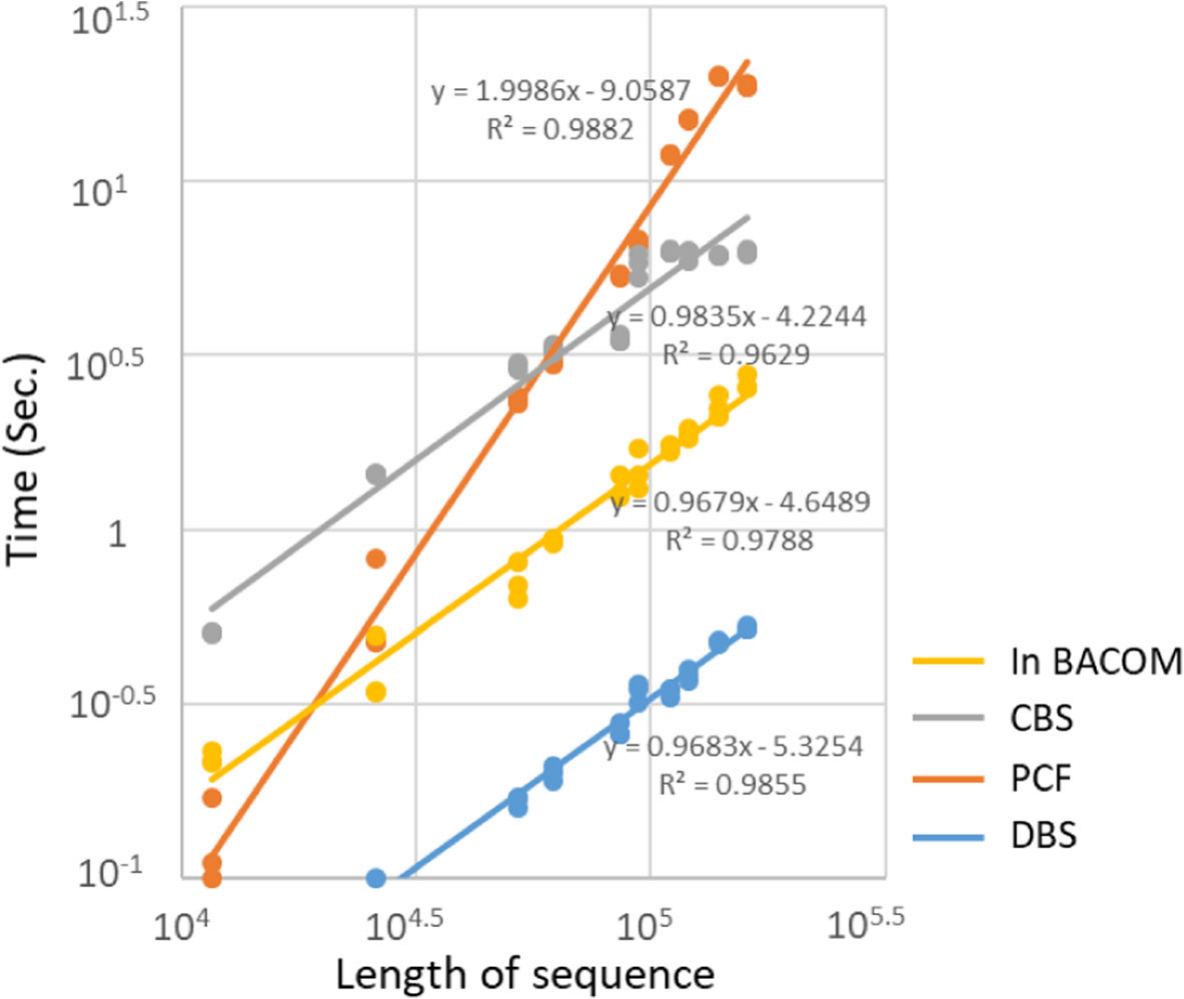
Computational complexity of time in the four algorithms. The solid lines with different colors represent the conventional linear regression models, which correspond to the data points with the same colors. The x-axis represents the logarithmic length of test samples (sequences), and the y-axis represents the logarithmic computation time

The computation time (in seconds) is shown for DNAcopy v1.52.0 (CBS), copynumber v1.18 (PCF), in BACOM, and DBS (ToolSeg) on the 26 K / 160 K simulation data set (10 samples) and on the values from an Affymetrix SNP 6.0 Array data set (10 samples). The slope of linear trend lines represents the computational complexity of each algorithm, which are shown in Fig. 5. All tests were performed on a PC with a 2.5GHz Intel i5 CPU with 8 GB of memory running Windows 10 and Java 8 (64-bit)

Next, we compare the computational complexity of time among the four above-mentioned methods using the actual computation time and length of the test samples. We select logarithmic transformation to reveal the relationship of computation time and sequence length. Using default parameter settings, we compared the computing times of DBS, CBS, PCF and the method in BACOM on the expanded samples in the 11.6 K ~ 160 K simulation data set. In Fig. 5, the data points with different colors correspond to the computation results of different algorithms in a logarithmic coordinate system. The conventional linear regression model corresponding to the data points of each method is shown as solid lines with the same colors. The slope of these lines represents the order of complexity in Big O notation, which is used to classify algorithms according to how their running time or space requirements grow as the input size grows. In Table 2, the slope of the linear regression of DBS, CBS and PCF all are approximately 1. These three algorithms benefit from the algorithm structure with nearly linear complexity, O(*n* ∙ log *n*). The slope of the method in BACOM is 2, because this method mainly contains a complete double circulation.

Further, the time complexity of the first phase in DBS is O(*n* ∙ log *K*), where *K* is the number of segments in the result of the first phase, and *n* is the length of observation sequence to be split. Because *n* ≫ *K*, the time complexity approaches O(*n*). At the second phase, the time complexity is O(*n* ∙ log *n*), because $\mathcal{W}$ is a geometric sequence with a common ratio of 2 by default. Although the time complexity of DBS is a mixture of O(*n*) and O(*n* ∙ log *n*) in theory, but the speed of DBS depends on the frequency upon triggering binary segmentation with various sliding windows. Thus, the speed of DBS is quite variable from sample to sample of the same length. As a result, the time complexity of DBS is basically no different than the other two algorithms (CBS and PCF) in the benchmarking. Just the speed of DBS is indeed larger than them. In Fig. 5, the slopes of the lines corresponding to the three algorithms are the same, but the line of DBS is at the bottom. In conclusion, DBS and other methods typically provide similar results and have equivalent accuracy; however, DBS enjoys a significant advantage in computation performance.

## Conclusions

We have developed a variant of binary segmentation based on least absolute errors (LAE) principles combined with heuristics using CLT that we call Deviation Binary Segmentation (DBS) for identifying genomic alterations in array copy number experiments. We have introduced a suite of platform-independent evaluation mechanisms based on the MLPA binary classifier as the gold standard. DBS was applied to a test dataset that has a similar data structure as a real case. The algorithm performs similarly to other leading segmentation methods in terms of sensitivity and specificity. In addition, the proposed algorithm can provide significant degrees of breakpoints in the results, and find breakpoint locations by searching for the extreme of test statistic. Furthermore, DBS benefits from the algorithm structure with a computational complexity of O(n*log n), which gives a further marked reduction in computation time using heuristics with trimmed first-order difference variance for searching for potential breakpoints. The proposed algorithm is easy to generalize and is computationally very efficient on high-resolution data. The Java Application offers a user-friendly GUI to the proposed algorithms and is freely available at https://gitee.com/w3STeam/ToolSeg.
